# Work Engagement among Acute Care Nurses: A Qualitative Study

**DOI:** 10.1155/2023/2749596

**Published:** 2023-10-17

**Authors:** Hind Al Mamari, Patricia S. Groves

**Affiliations:** ^1^Ministry of Health, P.O. Box 393, Muscat 100, Oman; ^2^University of Iowa College of Nursing, Iowa City, IA 52242, USA

## Abstract

**Aims:**

To understand how Omani nurses conceptualize work engagement, explore factors influencing engagement, and identify strategies to improve work engagement.

**Design:**

A qualitative study design.

**Methods:**

Semistructured interviews were conducted with twenty-one Omani nurses from four acute-care hospitals. Interview transcripts were examined using directed content analysis.

**Results:**

Participants defined work engagement as a positive state where nurses are engaged physically, emotionally, and mentally with work. Mentally engaged nurses' minds are occupied with patients even when they are off duty. Organizational factors affecting work engagement were leadership, teamwork, autonomy, pay, and job demand. Individual factors affecting engagement included considering nursing a rewarding profession. A social factor was family commitments. Strategies suggested to improve engagement included improved pay and monetary incentives, working system flexibility, open-door policy, performance feedback, recognition, and resources.

**Conclusion:**

Mentally engaged nurses are attached to work even when they are off duty. Nurses' gait and facial expressions can indicate high or low work engagement. Nurses with family obligations felt drained of energy, affecting their vigor and enthusiasm at work. *Implications*. Management skills and practices impact work engagement. Nurse's feedback can be used to improve practice and design interventions that promote nurses' engagement.

## 1. Introduction

Having disengaged nurses in any healthcare organization is very costly; a Press Ganey Associates study found that having one disengaged nurse can cost the organization $22,200 in lost productivity [1]. Generally, disengaged employees are more likely to have a low level of organizational commitment, low morale and productivity, and high turnover [2]. Highly engaged nurses are essential for any healthcare organization so organizations can enjoy a wide range of favorable organizational outcomes, maintain effectiveness, and reduce the cost of disengagement.

Work engagement supports productivity and retention, making it of great interest to scholars and practitioners in various disciplines ranging from business to healthcare [3, 4]. Nursing work engagement has captured the attention of nursing scholars and practitioners looking to resolve healthcare challenges such as high medical costs, worldwide shortage of healthcare workers, rising medical error rates, and increasing pressure to provide high-quality care [3]. Adding to those concerns, the nature of the nursing profession leads nurses to experience stress, burnout, high absenteeism, high turnover, and a low level of satisfaction, which negatively affects the nurses' work engagement [5]. To face these challenges, many organizations have asked their leaders to initiate and maintain a work engagement culture among their workforce [6].

## 2. Background

*Work engagement* is “a positive, fulfilling, work-related state of mind that is characterized by vigor, dedication, and absorption” [7]. *Vigor* is characterized by having high levels of energy and persistence while working and facing difficulties. *Dedication* refers to strong involvement in one's work along with experiencing feelings of enthusiasm, pride, and challenge. *Absorption* denotes full concentration and difficulties with detaching oneself from work.

The link between nursing work engagement and favorable organizational outcomes is well established. Compared to nurses who do not feel engaged, nurses who feel engaged seem to be more satisfied with their job [8, 9] and have less intention to leave their job [10]. Nurses who are more engaged in their work also demonstrate innovative behaviors [11] and affective organizational commitment [8].

Despite nursing scholars' and practitioners' increasing interest in work engagement, most work engagement studies were conducted in Western and European countries, and little is known about work engagement in other countries. As a result, our knowledge about factors influencing nurses' work engagement and strategies to improve nursing work engagement in Oman or similar contexts is limited. In particular, 96 articles in work engagement were reviewed by the principal investigator (PI), who determined that most of the nursing studies were conducted in European and Western countries. Some of the main geographical sources of European nursing work engagement literature were from Spain (e.g., [8, 12]), Belgium (e.g., [9, 13]), and Ireland (e.g., [14]). In regards to Western countries, many studies were conducted in the US (e.g., [15–17]) and some studies were conducted in Australia (e.g., [15]). The main geographical source of Eastern nursing work engagement literature was from China (e.g., [18]). There were also limited studies from Japan (e.g., [19]) and Malaysia (e.g., [20]).

Western and European countries have different economic systems, educational systems, and cultural values than eastern countries [4, 21]. Cultural variations and features could affect work engagement [4, 22]. In particular, factors, antecedents, and/or predictors of work engagement might be meaningful in one culture and not meaningful to another one [4].

No previous peer-reviewed studies were conducted in Oman; however, one study, conducted in Saudi Arabia, has a cultural context similar to Oman. This cross-sectional study examined work engagement among nurses and the relationship between work engagement and personal characteristics [23]. Investigators did not explore factors influencing work engagement or recommend strategies to improve nursing work engagement, preventing others in a similar cultural context from effectively utilizing the findings.

In addition, most work engagement studies have used quantitative methods [3]. Quantitative research provides valuable insight into nursing work engagement; however, these studies relied on self-report questionnaires and used standardized close-ended questionnaires in which respondents were unable to express their opinions and views. Qualitative studies allow researchers to explore in-depth factors influencing work engagement from the participants' points of view; participants might have their own views, thoughts, and experiences that could not be explained through standardized surveys and questionnaires. Participants might also explain aspects in work engagement that might be important in one nation and not important to another nation because of cultural variations. Qualitative research methods are used to describe life experiences from the perspective of the participants, not the perspective of the researchers [24], and are useful to understand meanings or knowledge constructed by people involved in the study [25]. Furthermore, using qualitative approaches might yield discoveries of “unexpected knowledge” that would be difficult to obtain through close-ended questions [26]. Qualitative studies are needed to provide in-depth knowledge on work engagement because individuals may ascribe different meanings to work engagement, and factors influencing an individual's work engagement may vary greatly.

The problem addressed by this study is culture, nursing, personal lives, and the healthcare system in Oman which are different than in other countries, and therefore, existing literature is inadequate to understand nursing work engagement in Oman. A qualitative study was needed to explore the phenomena of work engagement from the Omani nurses' perspective.

## 3. The Study

### 3.1. Aims

The aim of the study was to understand work engagement among Omani nurses working in acute-care hospitals. The research questions were as follows:

1. How do Omani nurses working in acute-care hospitals conceptualize work engagement?
2. What organizational, individual, and social factors affect the work engagement of Omani nurses working in acute-care hospitals?
3. What strategies do Omani nurses working in acute-care hospitals suggest to improve work engagement?

## 4. Methods

### 4.1. Theoretical Framework

The job demands-resources (JD-R) model was used to guide this study. The model directed the approach to understanding work engagement in Omani hospitals via both the research questions and the specific interview questions used to elicit data collection. The JD-R model consists of two specific sets of working conditions that predict employees' well-being regardless of the occupational setting: job demands and job resources [27]. *Job demands* refer to the physical, psychological, social, or organizational job features requiring continuous physical or psychological effort. *Job resources* refer to the job features that help reduce job demands, achieve work-related goals, and improve personal growth.

### 4.2. Design

This study used a qualitative design, which allows the researcher to make sense of the participants' world and their experiences in their natural settings; the researcher stays close to the data and produces a description of the participant's experience in language similar to the participant's own language [28, 29].

### 4.3. Study Setting and Sample

The study population was Omani nurses representing four acute-care hospitals in Oman. These hospitals are in different regions and provinces throughout Oman and provide various specialty services to the population such as general medicine, cardiology, general surgery, obstetrics and gynecology, pediatrics, ambulatory, and inpatient services. The Royal Hospital has a population of 668 Omani nurses, Sultan Qaboos Hospital has 304 Omani nurses, Sohar Hospital employs 502 Omani nurses, and Al Rustaq Hospital has a population of 380 Omani nurses [30]. Eligibility criteria included the following: (a) Omani nurses working in acute-care hospitals and (b) two or more years of experience in nursing. Generally, the sample size of qualitative studies ranges between 20 and 30 interviews [31]. Prior qualitative studies on work engagement among nurses reported a sample size of 20 [14]. PI thus aimed to recruit between 20 and 25 participants.

PI received authorization from the directors of nursing in the selected hospitals as well as Institutional Review Board approval in Oman and at the University of Iowa. After obtaining approval, PI distributed a recruitment flyer in Arabic and English languages. A four-week recruitment period following posting of the recruitment message in the selected hospitals was allowed. After the interview, participants were also asked to point out the flyer to other potential participants if they felt comfortable doing so. PI reviewed the exempt information sheet with potential participants over the phone and then sent it via an email link. The link to the exempt information sheet was sent using Qualtrics, an online survey application. The participants were instructed to read the exempt information sheet thoroughly by themselves and were given a week to think and make their decision about participation in the study and to ask the investigator if they have additional questions. Clicking in the link “I consent to participate, begin the study” after reviewing the exempt information sheet indicated that the participants were willing and agreeing to participate. The exempt information sheet was available in Arabic and English languages.

To avoid job repercussions, directors of nursing in the selected hospitals did not know who participated. Not only did the researcher call the participants directly to arrange for the interviews, but also the participants were assured that their immediate supervisor would not know their responses to the questionnaires and that their feelings towards the topic and their names would be kept confidential, and any information they provided would be anonymous.

### 4.4. Data Sources/Collection

Prior to data collection, a semistructured interview guide was developed and piloted with three nurses. The questions were developed using existing work engagement literature (e.g., [5, 14, 27]) and by consulting qualitative experts. PI conducted study interviews using the guide via a secure Skype for Business application; the interviews lasted for 60 minutes and were audio-recorded. The interviews were conducted in the participants' native Arabic language, allowing free expression of thoughts. Basic demographic and work-related information were collected via an online survey. The participants were identified by using numbers (e.g., P01) to preserve privacy and confidentiality. Recruitment and data collection were stopped when data reached saturation, that is, when no new ideas were suggested by participants.

### 4.5. Data Analysis

Directed content analysis as described by Hsieh and Shannon [32] was used to analyze the interview data. The steps were as follows: (1) transcription, (2) identification of key concepts or variables and determination of operational definitions/description, (3) reading transcripts and highlighting text, (4) coding, (5) examination of data, and (6) selection of quotes. Microsoft Word was used for manual coding.

### 4.6. Ethical Considerations

Ethical considerations of human subjects were maintained through Institutional Review Board (IRB) review, consent, and protection of the identifiable information. To ensure human subjects' protections, ethics committee's approval was obtained from the University of Iowa and the Ministry of Health in Oman. Consent was obtained from each participant before participation in the study, and the participants were informed that their participation was completely voluntary.

### 4.7. Rigor

Four typologies of validity were used in this study as follows: descriptive validity, interpretive validity, theoretical validity, and generalizability [33]. Strategies implemented to establish descriptive validity included recording interviews and ensuring accurate transcription and translation, not omitting information, regardless of understanding and/or perceived relevancy, and providing “simple counts” to support claims about the prevalence of ideas among participants. To establish the interpretive validity, PI employed strategies such as trying to understand the perspectives of the participants and the meanings participants attached to their words and phrases and providing verbatim quotes to accurately describe the participants' subjective viewpoints and meanings. For theoretical validity, PI avoided artificially interpreting data to fit a specific theory. To establish generalizability, thorough descriptions about the context of the research and the study participants and rich, thick descriptions addressing the aims are presented so readers can determine the applicability of these findings to their context.

## 5. Findings

### 5.1. Participants

Twenty-one Omani nurses working in four acute-care hospitals participated.  Table 1 displays participant demographics.

### 5.2. Data Analysis

Although some variations existed within participants' stories, analysis of the data revealed 11 categories and eight subcategories, which are summarized in Table 2.

#### 5.2.1. RQ1: How Do Omani Nurses Working in Acute-Care Hospitals Conceptualize Work Engagement?

Three categories were identified in answer to RQ1: definition of work engagement, signs of a nurse with high work engagement, and signs of a nurse with low work engagement.

*(1) Category One. Definition of Work Engagement*. Omani nurses working in acute-care hospitals conceptualized *work engagement* as a positive state where nurses are “engaged physically and mentally with work” (Participant no.16 [P16]). Work engagement is more likely to occur when nurses experience a sense of loving one's job, pride, satisfaction, achievement, importance, interest, belonging with their organization, and enthusiasm in their job.

*Physically engaged* nurses mean nurses exert more effort and energy than expected to accomplish work goals and perform extra job responsibilities. A physically engaged nurse was described by participants as a “hard worker” (P02), “energetic” (P13), “have enough energy to use it wisely” (P11), “accomplish his work and perform extra roles… my contributions to the workplace mean engagement” (P18), and “I can give as much as I can” (P09).

To the participants, *mentally engaged* meant nurses who are focused, immersed, and attached to work even when off duty, as this participant explained, “If I travel outside my country, I feel engaged with my work. It comes to my mind the patient in bed number one, what happened to him? Is he extubated or not?” (P01). Another participant gave the example, “if I see the unit is busy, I stay with them even if my duty is finished…My evening shift ends at 9:00 pm but sometimes you see my nursing documentation is until 11 pm or 10:45 pm…I do not mind” (P15).

*(2) Categories Two and Three. Signs of High and Low Work Engagement*. Participants explained high- and low-engaged nurses can be recognized by how they show interest and enthusiasm, perform tasks and responsibilities, and express attitude while coming to work.

First, nurses who have high work engagement are recognized by how they demonstrate interest and enthusiasm at work. The participants explained that highly engaged nurses are responsible, hard workers, committed, innovative, and interested in improving their units. One participant described highly engaged nurses as those who “contribute and give up to their best, they keep the work's interest above other interests, and it is the main priority when at work” (P19). Another participant gave the example: “They are focused on work and do not bring their personal life issues to work. You see highly engaged staff separate their personal life from work when they come to duty” (P13). Participants frequently discussed how highly engaged nurses are cooperative and willing to help, especially when the unit had an unexpected staff shortage or a disaster. Participants gave the examples that a highly engaged nurse would “agree to cover any sick leaves” (P10), “come to the unplanned duty if called even if he has a strong excuse not to come” (P04), and “say if you need any help in the unit, just call me… I am ready to come” (P21). However, many participants discussed that poorly engaged nurses were not interested in performing extra tasks or improving their areas; those nurses just attended their duty by completing mandatory working hours and go home. One participant described “I always hear them saying we work according to the amount of salary we earn” (P04).

Second, nurses with high work engagement were recognized by how they performed tasks and responsibilities. One study participant pointed out a highly engaged nurse “looks to his assignment and he is satisfied” (P14). Participants frequently discussed how highly engaged nurses will work with conscientiousness; for example, “highly engaged staff are providing care from their heart, it is not the matter that I have to finish my duty and go home” (P16). On the other hand, nurses with low work engagement are viewed as careless and irresponsible, as this participant explained, “the low-engaged nurse has no sense of responsibility at all…when I receive a patient from the previous shift, I find a lot of nursing care is not carried out, a lot of pending requests” (P21).

Finally, nurses with high work engagement are recognized by their attitude while coming to work. One participant described how they could detect high work engagement from another's “gait when coming to duty…he comes and you can tell, he will spend a lot of effort and give more” (P07), and someone being “active and energetic when they come to duty regardless of their personal issues at home” (P13). Another participant discussed the facial expression of nurses, saying “it is obvious that if I love the work, you will find me coming to duty smiling, talking with others and greeting others” (P04). In contrast, “low work engagement staff distribute negativity among others” (P08) and frequently complain. Two participants described how they could detect low work engagement staff: “not energetic…forced to come to duty” (P13) and “looks upset…comes to operation theater and puts his head down, walks slowly. Even if you try to make him laugh, it is difficult to see his teeth and his smile” (P03).

#### 5.2.2. RQ2: What Organizational, Social, and Individual Factors Affect the Work Engagement of Omani Nurses Working in Acute-Care Hospitals?

Three categories and eight subcategories emerged to answer RQ2 about factors that affected the work engagement of Omani nurses, as seen in Table 3.

*(1) Category One. Organizational Factors*. Five subcategories of organizational factors were identified as follows: leadership, teamwork and interprofessional relationship, autonomy, pay and monetary incentives, and job demand. Table 3 displays subcategories of organizational factors that affected nurses' engagement and representative data from the interviews.

 *(i) Leadership*. All participants discussed the attitudes, characteristics, and skills of charge nurses (*n* = 21). Examples of characteristics that enhanced work engagement were effective communication, effective human resource management, caring, flexibility, involvement, effective performance appraisal skills, fairness, and appreciation. Participants explained that charge nurse's positive attitudes not only boosted their work engagement but also impacted their intention to stay as this nurse participant pointed out: “she (charge nurse) is very helpful, supportive, has a sense of humor… she is one of us…Maybe because of her I am still there for 11 years and I did not ask for a transfer out” (P15).

Thirteen study participants discussed the importance of the charge nurse for their engagement, and eight participants described how poor charge nurse leadership lessened their level of work engagement as this participant explained:

*“… my energy for work is lower than before. My passion for work became low. There are some days when I do not want to go to work never…never…never…because I know there will be someone [charge nurse] at work who will make me upset (P16).”*

In addition, 14 participants discussed nursing administration's poor leadership practices. Ineffective human resource management practices were most discussed, including nursing shortage, maldistribution of staffing, ineffective staff transfer mechanism between acute-care hospitals and primary healthcare institutions, favoritism, and lack of nursing administration meetings with nurses. One participant explained how poor nursing administration attitudes and management practices impacted her work engagement: “these affect my work engagement because I will not give the best quality care to the patients if I have workload and high patient census and there is not enough support” (P02).

 *(ii) Teamwork and Interprofessional Relationships*. All study participants described how their work engagement level increased when they were able to work in teams and had good relationships with other healthcare professionals. One participant explained how teamwork impacts her work engagement: “I was excited to go to work because I have the best team ever …the good thing that makes me continue in the unit until now is the teamwork” (P02).

 *(iii) Autonomy*. Autonomy refers to how much freedom the participants had while carrying out nursing assignments. Likewise, all study participants explained job autonomy increased their level of work engagement as this participant described: “I feel when you are going to work and you have the autonomy to do the work, your engagement to work increases, you love your work, and you feel you are proud of what you are doing” (P15).

 *(iv) Pay and Monetary Incentives*. Twenty study participants indicated that their salary was not fair and acceptable compensation for their years of experience, nature of work, place of work, type of shift, and certificates. All participants who pursued higher education (*n* = 10) and/or worked in specialized/critical areas criticized making the same salary as general nurses who worked in primary healthcare institutions or nonspecialized areas. Many participants explained how unfair pay affected their enthusiasm for their job, such as this participant: “We have a lot of nurses who are burned out and asked for a transfer out of the hospital…you are working in ICU and there is no risk allowance” (P20).

 *(v) Job Demands*. Participants described job demands in their organizations that lessened their work engagement level. Job demands included workload (*n* = 21), shift (*n* = 11), and patient harassment and demands (*n* = 7). The factors contributing to workload discussed by participants were high number of patients (*n* = 21), shortage of nurses and healthcare professionals (*n* = 20), shortage of equipment and supplies (*n* = 13), performing nonnursing tasks (*n* = 6), ineffective human resource management (*n* = 5), and performing additional responsibilities (*n* = 2). All study participants expressed that their high workload and time pressure affected patient safety because they did not have time to perform safe and effective nursing care. One study participant discussed how workload affects his enthusiasm, “I have enthusiasm when I go to work. I go and I am not hassled or upset. This enthusiasm does not last for the whole shift, because of the workload I feel my enthusiasm becomes gradually low” (P17).

In a notable overlap with the social factors' category described next, all 10 married participants who worked rotating shifts described how their spouses preferred them not to work weekends, holidays, and night shifts, leading to conflict. Some married female participants expressed that their husbands were not happy about their work, particularly after having children, and pressured them to request changing their duty or unit to one with no night shifts. One participant described, “sometimes, he (husband) pressures me and tells me the kids are the main priority if they are sick or my kids have something. I have to request time off, ask my colleagues to exchange the duty…it affects my work…my thinking” (P20).

Many participants discussed how working in the night shift for a long time (i.e., more than ten or fifteen years) made them feel tired, exhausted, and sleepless and affected their engagement, as this participant explained with her experience with the night shift and on-call duty, “I will be stressed and sometimes not prepared to go to work…It is the most difficult time for my psychological and physical health, and the issue after the night shift was being in many road traffic accidents” (P10).

*(2) Category Two. Social Factors*

 *(i) Family Commitments*. Only one substantial subcategory of social factors emerged: family commitments (*n* = 14) to address RQ2. Family commitments refer to participants' responsibilities at home and their dedication to meet the needs of their children and other family members. Nurses with family commitments may occupy their thoughts at work with home responsibilities waiting for them when they finish their duty or have less enthusiasm for work in general. As this participant voiced:

*“We have a lot of workload (at work) and at the same time, I am thinking of my other responsibilities that are waiting for me after I finish my duty, especially if I need to clean my home or look after my small child if he is sick (P11).”*

*(3) Category Three. Individual Factors*. Two subcategories of individual factors were identified: rewarding profession and personal characteristics.  Table 4 lists subcategories for individual factors.

 *(i) Rewarding Profession*. All participants found nursing to be a rewarding profession, meaning participants had positive perceptions and feelings towards the nursing profession. The most rewarding aspects of the participants' nursing job were caring for patients, acquiring knowledge and skills, noticing an improvement in patients' condition, gaining rewards from God, hearing prayers from patients and their relatives, and being known by people for their excellent nursing care. The participants felt emotionally engaged and described feeling “proud,” “satisfied,” and “loved,” despite the challenges of their profession. This participant explained the rewards impacting their work engagement: “I am very effective in the community…I feel I made a fingerprint in my work that is known by everybody at work. This makes me very proud of myself as a nurse” (P12).

 *(ii) Personal Characteristics*. Many participants discussed personal characteristics that affected their level of work engagement (*n* = 17). The most common positive personal characteristics discussed by nurse participants were being energetic, hardworking, and competent. These characteristics impact participants' level of work engagement; acquisition of skills made them good advocates for patients and improved confidence level, vigor, and enthusiasm at work as this participant describes, “I go with enthusiasm for my work…I am trying to do something new…” (P12).

#### 5.2.3. RQ3: What Strategies Do Omani Nurses Working in Acute-Care Hospitals Suggest to Improve Work Engagement?

The top suggestions from participants were related to pay and monetary incentives (*n* = 20, 95%), hospital policies (*n* = 18, 86%), management practices and skills (*n* = 18, 86%), recognition (*n* = 14, 67%), and resources (*n* = 10, 48%).  Table 5 lists the categories for RQ3 and representative data from the interviews.

Improving pay and providing monetary incentives for nurses with specialization and higher education were the most common suggestions from participants to improve work engagement (*n* = 20). Most participants also suggested policy changes (*n* = 18), including (1) increasing staff of general nurses, physicians, other healthcare professionals, and support staff, (2) ensuring flexible working hours for working mothers with children by which experienced married nurses are exempted from or have limited night shift during shift rotations, and (3) in the absence of fair salary compensation for specialization, implementing a rotation system between specialized areas and hospital out-patient clinics or primary healthcare institutions.

Other strategies suggested by most participants were improving nursing and hospital administration's practices and skills (*n* = 18). The participants recommended regular hospital and nursing management rounds, an open-door policy, and effective performance feedback. More than half of the participants suggested improving recognition as a strategy (*n* = 14) and suggested this could simply be verbal: “no need for gifts, saying thank you verbally has a positive impact on staff and improves their work engagement” (P04). Other suggestions were written praise such as certificates and public awarding of appreciation. One participant working in ER explained the importance of simple appreciation: “for instance, if we faced a disaster in a day and everyone knows that, they should appreciate staff even with a simple certificate…Our nursing and hospital administration should pay attention to us” (P15).

Many participants suggested improving resources to improve their work engagement (*n* = 10), including (1) introducing a workplace wellness program for employees to increase productivity, improve health behaviors, and reduce stress; (2) providing a workplace nursery; and (3) providing enough equipment and consumable supplies.

## 6. Discussion

Omani nurses who work in acute-care hospitals have a conceptualization of work engagement consistent with early and contemporary models of work engagement that suggest work engagement has three different facets: cognitive engagement, emotional engagement, and behavioral engagement [34, 35]. However, having a sense of loving one's job, achievement, and belonging/affiliation with an organization are concepts that were not mentioned in the literature regarding emotional engagement conceptualization.

Two other findings notably differed from extant literature. Surprisingly, the participants described how mentally engaged nurses think about their patients even when off duty. Work engagement in the literature was defined as a positive state of mind that happens mostly at work. This suggests that researchers should not limit examination of work engagement to certain times and places. One possible explanation is a culture in nursing towards prioritizing patients and improving nursing care quality, which consequently leads to nurses thinking about work after working hours.

Second, nurse participants discussed how high- and low-engaged nurses can be recognized at work from how they express attitudes while coming to work. Participants explained that they judge the level of work engagement via nonverbal cues such as nurses' gait and facial expressions. This aspect of recognizing low and high work engagement in nurses was not found in current work engagement literature.

Organizational factors affecting Omani nurse work engagement were largely consistent with current literature, including leadership (e.g., [16]), teamwork (e.g., [15]), autonomy (e.g., [13]), pay (e.g., [14]), and workload (e.g., [36]). Many participants expressed that rotating shifts, particularly night shift, are difficult, with their energy and enthusiasm adversely being affected by these shifts. This finding is consistent with that of a study conducted by Rivera et al. [17], who indicated nurses who worked day shift were more engaged than nurses who worked evening and night shifts. The nurse participants discussed how unfair pay and monetary incentives negatively impacted their work engagement; previous studies have produced mixed findings on this aspect. One possible explanation is the Omani nursing system which is different from that of other countries (e.g., the United States); nurses employed by the Ministry of Health are all paid the same salary regardless of their specialization, education, work assignment, and workload.

Family commitment was the only substantial subcategory that emerged under social factors; this may be related to Omani culture. In this study, some nurses with family obligations were not focused at work and were mentally occupied with responsibilities waiting for them at home. Other nurses with obligations such as taking care of home and children felt drained in energy, which consequently affected their level of vigor and enthusiasm when they were at work. The Ministry of Health lacks family friendly policies that enable nurses to balance their families and work; most nurses work full-time, fixed rotating shifts with no available part-time work for mothers. Research about the impact of family commitments, home-work interference, and family-work life balance on work engagement is scarce in nursing literature and across other disciplines [37]. Only one nursing study was identified, finding that family-work conflict was positively related to absorption among Polish nurses [38]; this is inconsistent with the findings from the current study. However, the negative impact of the participant's family life on work was inversely associated with vigor, dedication, and absorption among Dutch medical residents [37].

In terms of individual factors, all participants found nursing to be a rewarding profession; this is consistent with a study conducted by Freeney and Tiernan [14], who explained nurses consider patients recovering as a personal reward that makes them feel motivated and dedicated to their work. Participants also discussed how personality characteristics such as being active, energetic, hardworking, and competent positively affected their level of work engagement. These factors are in line with the study by Pérez-Fuentes et al. [12], who found that consciousness and extraversion were positively related to work engagement among nurses.

This study made an important contribution to the literature; published studies exploring the strategies to improve work engagement from the participants' perspectives are very limited. Only one recent study aimed to examine factors hindering the appearance of burnout syndrome and inducing nurses to present work engagement [39]. Suggestions offered by Omani nurses working in acute-care hospitals consistent with suggestions mentioned in Sanclemente-Vinue et al.'s study were improving financial rewards, resources, and recognition. Additional suggestions offered by Omani nurses included the following: improving pay, wellness programs, job rotation, flexible work arrangements, and several hospital and nursing management skills and practices.

### 6.1. Strengths and Limitations of the Work

The qualitative methodological option was appropriate for this study because little is known about work engagement in Oman's health context. Using this approach allowed sensemaking by the participants of their world and their experiences in their natural settings and understanding of work engagement from the participants' point of view. However, despite the richness of data, generalizability of the findings is limited to Omani nurses from four acute-care hospitals. PI transcribed and translated the interviews, relying on her experience and fluency in English. However, PI provided verbatim translations to an experienced qualitative researcher when questions arose. In addition, it is possible that other investigators may have interpreted the qualitative data differently. These limitations were offset by the procedures used to ensure trustworthiness, as previously described.

### 6.2. Recommendations for Further Research

While this qualitative study provided insight into work engagement among Omani nurses working in acute-care hospitals, future studies are needed. First, experimental or nonexperimental intervention studies are needed to examine the effectiveness of strategies that could improve work engagement among Omani nurses. Examples of strategies and interventions that need to study its effectiveness on work engagement are financial and nonfinancial incentives, leadership training, job rotation, and flexible work arrangements. Second, replications of the study with Omani nurses working in primary healthcare institutions should be conducted to explore any differences in their perceptions regarding factors affecting work engagement and strategies to improve work engagement. Third, comparative studies might be needed to explore the impact of pay and incentives on work engagement in healthcare settings similar or different to Oman. Finally, longitudinal studies that allow monitoring nurses' over time might produce greater insight into the development and promotion of work engagement at the individual and team level. Further studies are needed to investigate the impact of family commitment and job rotation on work engagement as these two variables are rarely studied in nursing literature.

## 7. Conclusion

This qualitative study is the first study designed to explore work engagement among Omani nurses. This study provides foundational knowledge about how Omani nurses working in acute-care hospitals conceptualize work engagement and what organizational, social, and individual factors affect nursing work engagement in Oman. In addition, strategies suggested by the participants to improve work engagement provide a foundation for developing and implementing work engagement interventions in Omani hospitals. These findings thus support creation of a work environment that supports and encourages work engagement, consistent with the Oman Health Vision 2050 to improve the quality of healthcare services and create a positive work environment among healthcare professionals [40].

## Figures and Tables

**Table 1:** Participant demographics.

Variables	*n*	%
Sex		
Female	17	81
Male	4	19
Age		
25–34 years	13	62
35–44 years	8	38
Marital status		
Married	15	71
Single	6	29
Having children		
Yes	14	67
No	7	33
Highest nursing educational degree		
Bachelor of science in nursing	10	48
Nursing diploma	11	52
Postbasic diploma or specialized training certificate		
Yes	10	48
No	11	52
Type of shift		
Three shifts	11	52
Morning shift only	10	48

	Mean (range)	SD

Years of nursing experience	12.7 (4–22)	5.1
Years of experience in current department	8.8 (6 months–20 years)	5.6

**Table 2:** Research questions, categories, and subcategories for work engagement conceptualization, factors, and strategies.

Research questions	Category	Subcategory
RQ1: How do Omani nurses working in acute-care hospitals conceptualize work engagement?	(i) Definition	
	(ii) Signs of a nurse with high work engagement	
	(iii) Signs of a nurse with low work engagement	

RQ2: What organizational, social, and individual factors affect the work engagement of Omani nurses working in acute-care hospitals?	(i) Organizational	(i) Leadership
		(ii) Teamwork and interprofessional relationships
		(iii) Autonomy
		(iv) Pay and monetary incentives
		(v) Job demand
	(ii) Social	(i) Family commitments
	(iii) Individual	(i) Rewarding profession
		(ii) Personal traits

RQ3: What strategies do Omani nurses working in acute-care hospitals suggest to improve work engagement?	(i) Pay and monetary incentives	
	(ii) Hospital policies	
	(iii) Management practices and skills	
	(iv) Recognition	
	(v) Resources	

**Table 3:** Subcategories about organizational factors affected the work engagement of Omani nurses working in acute-care hospitals.

Category	Subcategory	Description	Examples
Organizational	(i) Leadership	Negative and positive comments about charge nurses' and nursing administrations' decisions, management, and communication	“Our charge nurse is very supportive”
			“She is not good at distributing staff”
			“She shouts at us”
			“Nursing administration is only there for punishment”
	(ii) Teamwork and interprofessional relationship	Participants described the team in the unit, friends at work, and relationships with other healthcare workers	“I have the best team ever and the staff are excellent”
	(iii) Autonomy	Comments about how much freedom the participants had while carrying out nursing assignments	“I feel I have the full autonomy to do my work and I am not restrained”
	(iv) Pay and monetary incentives	Comments about low pay and unfair or absence of incentives	“There is unfairness, the salary is the same for those nurses with BSN and those with a nursing diploma”
	(v) Job demand	Comments about nursing practice environment's stressors that required participants to perform extra efforts to perform duties and achieve work goals	
	(vi) Workload	Comments about having more work than the participants can perform and should be accomplished in a specific time	“We are short of 13 staff nurses to do what we supposed to do”
	(vii) Shift	Comments about the rotating shifts	“The difficult thing is the night shift”
	(viii) Patient harassment and demands	Comments about behaviors and attitudes expressed by patients or their relatives to nurse participants at work	“Some patients come for fighting and disturb your mood especially if the shouting was in front of everybody”

**Table 4:** Subcategories about individual factors affected the work engagement of Omani nurses working in acute-care hospitals.

Category	Subcategory	Description	Examples
Individual factor	Rewarding profession	Nurse participants' perceptions about what nursing means to them and the most rewarding aspects of the job	“The best thing in my work is serving patients and the community”
			“I am very proud to be a nurse. Although, it is not my dream to be a nurse…”
	Personal traits	Comments about quality and characteristics of participants	“I am active…”
			“I am a knowledgeable and skillful nurse in this place, and they trust me”

**Table 5:** Categories for suggestions to improve work engagement among Omani nurses working in acute-care hospitals.

Category	Description	Examples
Pay and monetary incentives	Suggestions to improve pay and provide monetary incentives	“They need to consider my post basic diploma and pay me more”
		“MOH has to give risk allowance for nurses working in critical areas”

Hospital policies	Suggestions to develop new policies or review and implement current policies	“Rotation between primary healthcare nurses and ER nurses and other nurses in the hospital”

Management practices and skills	Suggestions to improve nursing management practices	“The administration should open doors for discussion and listen to us”

Recognition	Suggestions to provide verbal and written praise for nurses' valuable efforts and contributions	“A word of appreciation can affect you positively. We need verbal rewarding”

Resources	Suggestions to improve hospital resources	“Nursery near the hospital”
		“Provide enough equipment”

## Data Availability

The data used to support the findings of this study are included within the manuscript.
